# Tracking type specific prevalence of human Papillomavirus in cervical pre-cancer: a novel sampling strategy

**DOI:** 10.1186/1471-2288-12-77

**Published:** 2012-06-14

**Authors:** Edward K Waters, John Kaldor, Andrew J Hamilton, Anthony MA Smith, David J Philp, Basil Donovan, David G Regan

**Affiliations:** 1 The Kirby Institute, The University of New South Wales, Sydney, NSW, 2052, Australia; 2 Melbourne School of Land and Environment, The University of Melbourne, Dookie College, Dookie, Victoria, Australia; 3 Australian Research Centre in Sex, Health and Society, La Trobe University, Melbourne, Victoria, Australia

## Abstract

**Background:**

Surveillance designed to detect changes in the type-specific distribution of HPV in cervical intraepithelial neoplasia grade 3 (CIN-3) is necessary to evaluate the effectiveness of the Australian vaccination programme on cancer causing HPV types. This paper develops a protocol that eliminates the need to calculate required sample size; sample size is difficult to calculate in advance because HPV’s true type-specific prevalence is imperfectly known.

**Method:**

A truncated sequential sampling plan that collects a variable sample size was designed to detect changes in the type-specific distribution of HPV in CIN-3. Computer simulation to evaluate the accuracy of the plan at classifying the prevalence of an HPV type as low (< 5%), moderate (5-15%), or high (> 15%) and the average sample size collected was conducted and used to assess its appropriateness as a surveillance tool.

**Results:**

The plan classified the proportion of CIN-3 lesions positive for an HPV type very accurately, with >90% of simulations correctly classifying a simulated data-set with known prevalence. Misclassifying an HPV type of high prevalence as being of low prevalence, arguably the most serious kind of potential error, occurred < 0.05 times per 100 simulations. A much lower sample size (21–22 versus 40–48) was required to classify samples of high rather than low or moderate prevalence.

**Conclusions:**

Truncated sequential sampling enables the proportion of CIN-3 due to an HPV type to be accurately classified using small sample sizes. Truncated sequential sampling should be used for type-specific HPV surveillance in the vaccination era.

## Background

Infection with human Papillomavirus (HPV) is recognised as the main cause worldwide of both cervical cancer [1-3] and its precursor lesions, cervical intraepithelial neoplasia grades 2 and 3 (CIN-2, 3) [4]. In 2007, following the development of highly efficacious vaccines against oncogenic HPV subtypes 16 and 18 [1,5,6], Australia became the first country in the world to embark on a national vaccination program [7,8], and other countries have now begun various forms of program.

Ultimately, the success of such programs will be measured by the extent to which they reduce cancer incidence, but shorter term changes in benign and pre-cancerous lesions caused by HPV can be used to assess their impact [7]. In Australia, reductions have been reported in the incidence of CIN-3, [9] and genital warts [10]. Warts are caused by HPV types 6 and 11, which the quadrivalent vaccine protects against in addition to HPV 16 and 18 [1].

Key indicators in the early evaluation of an HPV vaccination program are provided by monitoring the distribution of HPV types. It is important both to track the expected decline in prevalence of the types that the vaccine protects against, and to monitor the prevalence of oncogenic types that are not the target of current vaccines [8]. However, planning surveys of HPV type distribution is challenging because current prevalences are only known imprecisely, so sample size estimates cannot be made with any confidence. It is therefore difficult to plan logistical and resource requirements. In order to address this problem, we developed a sequential approach to survey design, with particular application to the monitoring of HPV type distribution in CIN-3.

## Methods

### Sampling strategy

The sequential sampling approach to monitoring the prevalence of particular characteristics in a population has been used extensively in agriculture and industry, as well as in public health, where lot quality assurance sampling methods are particularly important [11-18]. We developed an approach based on the truncated sequential sampling method used for monitoring drug resistance in HIV infection [17]. Truncated sequential sampling differs from the Wald sampling method used in agriculture and industry by stopping (truncating) sampling when a classification of moderate prevalence can be made; normally, sequential sampling only allows classifications of high and/ or low prevalence [12,15,17]. The underlying strategy is to start with a limited number of specimens (*N*_*min*_), make an initial estimate of prevalence of the characteristic of interest (in this case, the proportion positive for a specific HPV type), and then continue to obtain specimens and recalculate the prevalence estimate until predetermined statistical criteria have been met.

**Figure 1:**
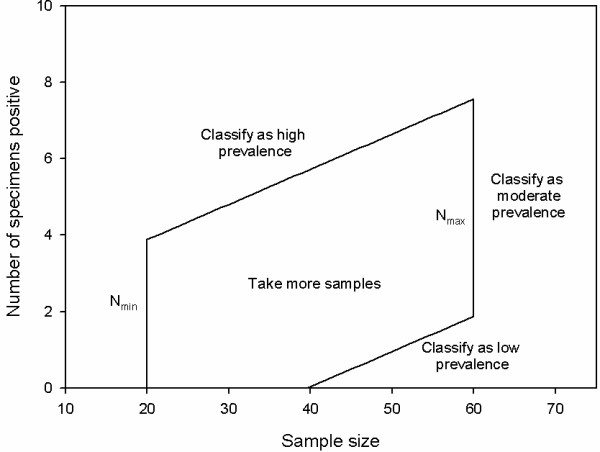
**Graphical representation of the truncated sequential sampling plan.** Classifications of high and low prevalence are based on breaching the upper or lower stop lines after the starting sample size (*N*_*min*_) has been collected. Sampling continues when the proportion of the sample positive for an HPV type falls between the lines. A classification of moderate prevalence is made and sampling is stopped (truncated) when the maximum sample size (*N*_*max*_) has been collected.

The specific criteria are that the estimate can be classified as coming from a population with a low, moderate or high prevalence of an HPV type with an acceptably low probability of being a false positive or a false negative [15]. Real world data are used to define thresholds used to classify prevalence as low, moderate or high [17]. Stopping rules, based on these thresholds and the probability of a false positive or false negative are used to determine whether a classification of high or low prevalence can be made (sampling should stop) with an acceptable probability of error or whether sampling should continue. If a predefined maximum sample size is collected and a classification of high or low prevalence has not been made, prevalence is classified as moderate.(*N*_*max*_) Stopping rules are commonly represented visually as pairs of lines on a graph delineating an area which, if breached, mean that sampling should be stopped and a classification made (see Figure 1) [15].

The *y* intercepts for the upper and lower stop lines in Figure 1*c*_*upper*_ and *c*_*lower*_, and the common slope (*m*) were calculated using Wald’s formulae [17,18,20]:

(1) cupper=ln1−βαlnp2q1p1q2

(2) clower=lnβ1−αlnp2q1p1q2

(3) m=lnq1q2lnp2q1p1q2.

In equations [1-3]*α* and *β* are the nominal probabilities of a false positive and false negative, *p*_*1*_ and *p*_*2*_ are thresholds defining low and high prevalence and *q*_*1*_ and *q*_*2*_ are equal to 1 - *p*_*1*_ and 1 - *p*_*2*_[17]. The maximum sample size, *N*_*max*_, used for truncation is determined arbitrarily [17].

### Applying the strategy to CIN-3

The sequential sampling approach can be used in any population, but in the context of evaluating the impact of HPV vaccination, it is most relevant for CIN-3. Monitoring HPV prevalence in normal cervical specimens, or lower grade CIN, is known to detect a high proportion of infections that are transient or lesions that will regress [19], and are therefore unlikely to cause cancer, while the time to develop invasive cancer is too long to detect changes due to vaccination.

We used thresholds of (a) less than 5% for an HPV type, designating low prevalence; ( b) 5-15% positive, designating moderate prevalence; and ( c) greater than 15% positive, designating high prevalence. In Australia only HPV 16 may occur in more than 15% of CIN-2 and 3; other types are evenly divided between those associated with ≤5% (HPV 52, 39, 68, 66 and 82) and 5 -15% (HPV 18, 33, 31, 58 and 73) prevalence in CIN-2 and 3 specimens [3]. We employed nominal false positive (*α*) and false negative (*β*) probabilities of 0.01 and 0.025, exceeding commonly used standards of α = 0.05 (statistical significance) and 1-*β* = 0.8 (statistical power).

Based on prior experience in other fields, a starting sample size (*N*_*min*_) of 20 specimens was considered large enough to make a classification in many cases. The choice of starting sample size does not affect the eventual classification of prevalences [17]. We used a maximum sample size for truncation of three times the minimum sample size (*N*_*max*_ = 60). Myatt and Bennett [17] trialled maximum sample sizes of 25–60 when testing the truncated sampling method, and found that a sample size of 47 was adequate for a plan using similar thresholds, so a maximum sample size of 60 was expected to provide an appropriate balance between accuracy and sampling intensity.

### Assessment of the sampling plan for CIN-3

We used simulation to assess how well the sequential sampling approach worked in classifying HPV types as follows. First, we generated synthetic data representing samples of CIN-3 cases, each with a different proportion positive for an HPV type). We generated 1,000 data sets under two different scenarios: one in which all prevalences between 0.00 and 1.00 were equally likely (uniform distribution); and another in which the most likely prevalence (mode) was 0.05, or 5%, with a linear decay to 0.00 at the lower limit and 1.00 at the upper limit (triangular distribution). The use of an upper limit of 1.00 is legitimate because very large proportions of some samples may be positive for high prevalence types – HPV 16 in particular has been detected in up to 95% of specimens in some studies [2]. The use of a triangular distribution is intended to simulate the most frequent sampled value for the prevalence of an HPV type being low (0.05). At present most types other than HPV 16 and 18 occur in 0-10% of samples so this distribution might capture the most likely value for positivity to most types [2,3]. We then used the sampling plan to simulate the process of sequential sampling 10,000 times from each of the data sets. Each one of these 10,000 implementations of the sampling plan is referred to individually as a resembling iteration. The code for simulating the implementation of the sampling plan was originally written in R ( http://www.r-project.org) but for this paper the code was translated into plain C for improved efficiency.

For each of the 1,000 data sets, we summarised the result of the 10,000 resampling iterations by first calculating the proportion of times the prevalence was classified as low, medium or high (designated PC_low_, PC_mod_ and PC_high_) and the sample size required to make each classification. PC_low_, PC_mod_ and PC_high_ were then plotted against the true prevalence in each data set, to show the relationship between the probability of classifying a data-set as being either of low, moderate or high positivity and the true proportion positive in the data set. Variation in the sample size collected was summarised using tables. Graphs were created using SigmaPlot 8.0 (Systat, Richmond, CA, USA).

The rate at which incorrect classifications were made over all sampling iterations was also calculated. Incorrect classifications were sub-categorised as incorrect classifications of low, moderate or high prevalence. Gross misclassifications are those in which a sample with truly high prevalence is classified as having low prevalence, or vice versa [17]. The rate at which each of these types of gross misclassification occurred was also recorded and the results tabulated.

## Results and discussion

**Figure 2:**
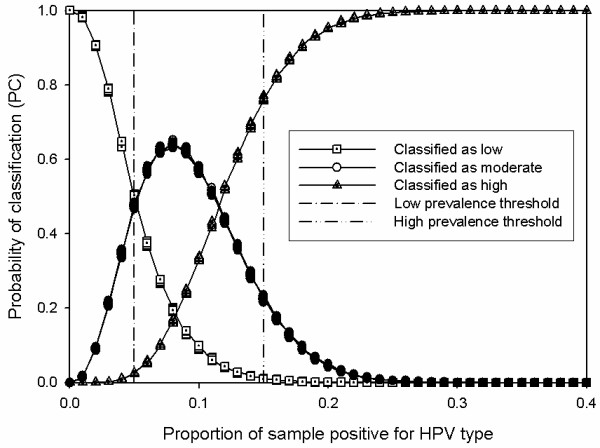
**Probability of classifying prevalence as low, moderate, or high over 10,000 resampling iterations over1,000 data sets with true prevalence (the*x*axis) sampled from a uniform distribution.** The dashed vertical lines represent the classification thresholds for low and high prevalence.

The sequential sampling strategy resulted in correct classification of HPV prevalence in > 90% of resampling iterations (see Table 1). Figure 2 shows that over 10,000 resampling iterations on a data-set in which the proportion positive for HPV type was 0.001 (i.e. clearly low prevalence) or 0.55 (clearly high prevalence), the sampling strategy always led to a correct classification. In a more difficult case, for example where the positive proportion was very close to the high prevalence threshold (0.149), the sequential strategy still performed very well, returning a classification of moderate prevalence on ~23% of occasions, of high prevalence on ~77% and of low prevalence almost never. The results shown in Figure 2 differed little whether the plan was employed on data-sets across which HPV positivity was distributed uniformly or where positivity was most frequently around 5% (triangular distribution). Therefore, for clarity of interpretation of Figure 2, only curves and scatter from the uniform distribution scenario are presented.

**Table 1:** Rate and type of incorrect classifications during all sampling iterations

**Distribution of HPV positivity**	**Any incorrect classification**		**Gross misclassification**		
	**Misclassified as**	**Misclassifications per 100 sampling iterations**	**True prevalence**	**Misclassified as**	**Misclassifications per 100 sampling iterations**
	Low	1.24	High	Low	1.31*10^-2^
Uniform	Moderate	1.23	Low	High	1.49*10^-2^
	High	3.91			
	Low	2.21	High	Low	1.92*10^-2^
Mode = 0.05	Moderate	2.04	Low	High	3.47*10^-2^
	High	6.1			

The most serious kind of gross misclassification is arguably when a plan classified a sample as having a “low” prevalence when it was in fact “high”. This type of error occurred very rarely (less than 0.05 times per 100 resampling iterations – see Table 1). Serious classification errors occurred more often under the scenario where prevalences were clustered around 0.05 (triangular distribution) than under the uniform scenario because a higher proportion of data sets had HPV positivity between 0 - 20% because of the peaked nature of the sampling distribution (35% vs.18%). These types of errors were still extremely rare however, also occurring less than 0.05 times per 100 resampling iterations (see Table 1).

A much higher sample size was required to classify samples with low prevalence than high prevalence (see Table 2). This is not unexpected given the negative intercept of the lower stop line (see Figure 1), which mandates that if prevalence is low a substantial number of samples are required to determine this. The sample size required to make a classification of moderate prevalence could obviously be reduced by arbitrarily lowering the value of *N*_*max*_, but little would be gained by doing so as it would result in more opportunities of misclassifying samples of low prevalence as having moderate prevalence (inspect Figure 1). These results suggest that the plan could be most efficiently employed by using it only to monitor HPV types that are more common.

**Table 2:** Mean sample size collected over 10,000 resampling bouts from 1,000 simulated data sets with uniformly distributed HPV positivity, and over 10,000 resampling bouts from 1,000 data sets with a triangular distribution of HPV positivity

**Distribution of HPV positivity in data**	**True prevalence**	**Sample size collected to classify proportion positive for HPV type**	
		**Mean**	**St. dev.**
	Low	40.76	5.73
Uniform	Moderate	48.27	4.51
	High	21.29	3.46
	Low	43.37	4.83
Mode = 0.05	Moderate	48.91	4.22
	High	22.51	4.54

In practical applications of this method, pathology services could prepare specimens from cervical samples and send them to a suitable reference laboratory [7]. After analysis at the reference laboratory, proportions positive for given HPV types would be recorded; on reaching the starting sample size, the number positive for an HPV type would be compared to the stop chart in Figure 1, and a determination made about whether more specimens were required. Though a strict application of sequential sampling would require a single additional specimen be analysed if required, it would be more practical to increment by five. This would not fundamentally undermine the sequential sampling approach, but it could reduce its efficiency by resulting in more samples being analysed than required.

The use of the resampling method for validation has some affinities with how the plan might be used in reality, as the plan might well be employed multiple times on one sample to classify positivity of multiple HPV types without a loss of precision, providing the risk of infection with one HPV type is not affected by infection with another, as appears to be the case [20,21]. It should be noted that random sampling is not assumed or required in sequential sampling [14,15].

## Conclusion

Truncated sequential sampling represents a practical scheme for conducting type-specific surveillance of HPV in pre-cancerous lesions. It eliminates the need to accurately calculate a priori sample size and makes no assumptions about current HPV type distribution. During resampling, it classified prevalence of an HPV type with > 90% accuracy. This is not unexpected as the plans utilised a far higher statistical power (nominal type II errors of 2.5%) than implied by the 20% type II error rate often used as a benchmark in sample size calculations. A much lower average sample size (21–22 versus up to 48) was required to accurately classify high prevalence samples. Truncated sequential sampling represents a practical, efficient method of conducting HPV type-specific surveillance in the absence of accurate, prior information about type specific prevalence, and is especially efficient at classifying the likely prevalence of more common HPV types.

## Competing interests

Linkage Project (LP0883831) (DGR, DJP, AMAS, JK, and EKW) includes contributions from CSL Biotherapies Ltd (which distributes the quadrivalent HPV vaccine in Australia) and Victorian Cytology Service Inc, which monitors cervical screening in Victoria and the National HPV Vaccination Register. BD has received honoraria from CSL Biotherapies and is a member of an advisory board for GlaxoSmithKline and CSL Biotherapies. AJH has no conflicts of interest to declare.

## Authors contributions

EKW devised the research idea, conducted and analysed the computer simulations, and wrote the manuscript. AJH, AMAS, DJP and DGR assisted with the development of methods and edited the manuscript. JK and BD provided epidemiological advice on the biology of HPV infection and current surveillance protocols and edited the manuscript.

## Grant support

This paper was funded from the following sources: the Australian Government Department of Health and Ageing; Australian Research Council (ARC) Linkage Project (LP0883831) which included contributions from CSL Ltd and Victorian Cytology Service Inc. The views expressed in this publication do not necessarily represent the position of the Australian Government.

## Pre-publication history

The pre-publication history for this paper can be accessed here:

http://www.biomedcentral.com/1471-2288/12/77/prepub
